# A novel approach to calculating the thermic effect of food in a metabolic chamber

**DOI:** 10.14814/phy2.12717

**Published:** 2016-02-23

**Authors:** Hitomi Ogata, Fumi Kobayashi, Masanobu Hibi, Shigeho Tanaka, Kumpei Tokuyama

**Affiliations:** ^1^ Faculty of Health and Sport SciencesUniversity of TsukubaTsukubaIbarakiJapan; ^2^ Research Fellow of the Japan Society for the Promotion of ScienceTokyoJapan; ^3^ Graduate School of Comprehensive Human ScienceUniversity of TsukubaTsukubaIbarakiJapan; ^4^ Health Care Food Research LaboratoriesKao CorporationTokyoJapan; ^5^ Department of Nutritional ScienceNational Institute of Health and NutritionTokyoJapan

**Keywords:** Integrated physical activity, nonexercise activity thermogenesis, thermic effect of food, whole‐body indirect calorimetry

## Abstract

The thermic effect of food (TEF) is the well‐known concept in spite of its difficulty for measuring. The gold standard for evaluating the TEF is the difference in energy expenditure between fed and fasting states (ΔEE). Alternatively, energy expenditure at 0 activity (EE_0_) is estimated from the intercept of the linear relationship between energy expenditure and physical activity to eliminate activity thermogenesis from the measurement, and the TEF is calculated as the difference between EE_0_ and postabsorptive resting metabolic rate (RMR) or sleeping metabolic rate (SMR). However, the accuracy of the alternative methods has been questioned. To improve TEF estimation, we propose a novel method as our original TEF calculation method to calculate EE_0_ using integrated physical activity over a specific time interval. We aimed to identify which alternative methods of TEF calculation returns reasonable estimates, that is, positive value as well as estimates close to ΔEE. Seven men participated in two sessions (with and without breakfast) of whole‐body indirect calorimetry, and physical activity was monitored with a triaxial accelerometer. Estimates of TEF by three simplified methods were compared to ΔEE. ΔEE, EE_0_ above SMR, and our original method returned positive values for the TEF after breakfast in all measurements. TEF estimates of our original method was indistinguishable from those based on the ΔEE, whereas those as EE_0_ above RMR and EE_0_ above SMR were slightly lower and higher, respectively. Our original method was the best among the three simplified TEF methods as it provided positive estimates in all the measurements that were close to the value derived from gold standard for all measurements.

## Introduction

Daily total energy expenditure comprises three principal components: the basal metabolic rate (BMR), energetic cost of physical activity, and thermic effect of food (TEF). BMR measurement is unambiguous by definition, it is the postabsorptive metabolic rate measured in a supine position in the morning. The energetic cost of physical activity can be separated into two components: exercise‐related activity thermogenesis and nonexercise activity thermogenesis (NEAT) (Levine et al. 1999; Levine 2007). The TEF is associated with the digestion, absorption, and storage of food, and is believed to have both facultative and fixed components (Levine 2005). Moreover, the magnitude of the TEF may differ between lean and obese individuals (Reed and Hill 1996), and the TEF lasts for several hours (Belko et al. 1986; D'Alessio et al. 1988; Kinabo and Durnin 1990; Reed and Hill 1996), during which NEAT also contributes to the total energy expenditure. Therefore, although the differences among the three principal components of energy expenditure are conceptually clear, additional effort is required to quantitate each component, particularly NEAT and the TEF (Levine 2002).

The TEF can be calculated by two different approaches. Tataranni et al. proposed computing the difference in the 24‐h energy expenditure between the fed and fasting states (ΔEE) (Tataranni et al. 1995); some consider this method the gold standard for estimating the TEF. However, this method requires two separate measurements. Alternatively, Schutz et al. proposed estimating the TEF as the difference between the postabsorptive resting metabolic rate (RMR) and the energy expenditure at 0 activity (EE_0_), which was estimated from the intercept of the linear regression between energy expenditure and physical activity in the postprandial state (Schutz et al. 1984). This approach and its modified version are commonly used (Westerterp et al. 1999), which involves subtracting the sleeping metabolic rate (SMR) instead of the RMR. However, their accuracy has been questioned because of their poor reproducibility and frequent underestimation, leading to negative TEF values (Ravussin et al. 1986; Westerterp et al. 1999).

It is well known that physical activity is not immediately reflected as changes in energy expenditure (LaForgia et al. 2006) or body temperature (Refinetti and Menaker 1992). Weinert et al. successfully related physical activity to changes in body temperature by introducing the concept of integrated physical activity over a specific time interval (Weinert and Waterhouse 1998). Using their approach, the highest correlation coefficient between body temperature and locomotor activity integrated over a varying time period was determined in mice. Thus, the activity‐related increase in body temperature was subtracted from the observed time course of body temperature, allowing the circadian rhythm, which is free from the effects of locomotor activity, to be evaluated. Based on this strategy, we propose a novel method for calculating the TEF by removing NEAT from the time course of postprandial energy expenditure. Various methods of TEF calculation have been proposed, but there is no calculation method focused on this point. This strategy may also allow the relationship between physical activity and energy expenditure to be assessed, thus providing an estimate of the energetic cost of physical activity.

Our original method for calculating the TEF involves the following steps: (1) evaluate linear dependency of energy expenditure on integrated physical activity over a given time interval accounting for a prolonged increase in energy expenditure after physical activity; (2) estimate NEAT from its linear dependency on integrated physical activity; (3) construct a time course of energy expenditure free from NEAT; and (4) calculate postprandial increase in energy expenditure free from NEAT as the TEF. In the present validation study, we compared three simplified methods of TEF estimation against Tataranni's method (ΔEE) (Tataranni et al. 1995). We selected three methods that take account of the increase in energy expenditure due to physical activity: Schutz's method (EE_0_ above the RMR) (Schutz et al. 1984), a modified version of Schutz's method (EE_0_ above the SMR) (Westerterp et al. 1999), and our original method (energy expenditure free from NEAT above the preprandial value). We also aimed to identify which calculation methods return positive value for TEF estimates as well as estimates close to the gold standard method (ΔEE) (Tataranni et al. 1995).

## Methods

### Subjects

Seven healthy young men (mean ± SD age: 24.7 ± 2.9 years; height: 177.8 ± 7.3 cm; body weight: 73.6 ± 12.1 kg; body fat percentage: 18.9 ± 5.4%; body mass index: 23.2 ± 2.8 kg/m^2^) participated in this study. The subjects habitually ate breakfast and had no chronic diseases that could affect energy metabolism or daily physical activity. We informed the subjects about the experiments and their associated risks. This study was approved by the Local Ethics Committee of the University of Tsukuba, and all subjects provided written informed consent to participate.

### Study protocol

The subjects participated in two trials: with breakfast (i.e., three‐meal condition) and without breakfast (i.e., two‐meal condition). The subjects stayed in a room‐sized respiratory chamber for 33 h. Exercise was prohibited and the subjects were asked to remain seated during this period. The two trials were conducted 1 week apart with a randomized repeated measures design. After entering the chamber at 2200 h, the subjects slept for 8 h from 2300 h to 0700 h the next morning, and 24‐h energy metabolism was evaluated (from 0700 h to 0700 h the following morning). If the subjects awoke during the sleep period, they were required to stay on the bed without moving around the room. During the indirect calorimetry, the subjects consumed breakfast (0800 h, 689 ± 121 kcal) or no breakfast (0 kcal), lunch (1200 h, 761 ± 115 and 1105 ± 172 kcal for the three‐ and two‐meal conditions, respectively), and dinner (1900 h, 741 ± 130 and 1085 ± 184 kcal, for the three‐ and two‐meal conditions, respectively). When breakfast was not provided, the subjects ate larger meals at lunch and dinner such that the 24‐h energy intake was the same for both dietary conditions. The meals given during the calorimetry were individually adjusted (2190 ± 354 kcal/day, 17% protein, 21% fat, and 62% carbohydrates) based on the estimated energy requirements and normal macronutrient balance for Japanese adults (Ministry of Health, Labour and Welfare of Japan 2010).

### Measurements

#### Physical activity

Each subject wore a triaxial accelerometer (30 mm depth × 80 mm width × 17 mm height, 19 g; TANITA Co., Ltd., Tokyo, Japan) in his left shirt pocket throughout the whole‐body indirect calorimetry period. The triaxial accelerometer measures three‐dimensional accelerations (count/min), and can accurately estimate ambulatory and normal household activities (Brage et al. 2007). Each of the three signals from the triaxial accelerometer was passed through an analog low‐pass filter (cut‐off, 730 Hz) and digitized. After removing the gravitational acceleration component from the signal, the synthetic acceleration of all three axes (i.e., vector magnitude) was calculated as a measure of physical activity for a given interval (1 min). According to the manufacturer, the sensitivity of the accelerometer is 2.4 *μ*G/count.

#### Whole‐body indirect calorimetry

Indirect calorimetry with an improved transient response was performed as described in our previous studies (Katayose et al. 2009; Tokuyama et al. 2009). Briefly, the dimensions of the airtight chamber for the whole‐body indirect calorimeter are 3.45 m width × 2.00 m depth × 2.10 m height, with an internal volume of 14.49 m^3^ (FHC‐15S, Fuji Medical Science Co., Ltd., Chiba, Japan). We precisely measured the concentrations of O_2_ and CO_2_ both in the incoming‐ and outgoing air using online process mass spectrometry (VG Prima *δ*B, Thermo Electron Co., Winsford, UK). O_2_ consumption and CO_2_ production rates were calculated every minute using an algorithm for the improved transient response. Measurements of energy expenditure were advanced by 2 min, taking into account the response of open‐circuit indirect calorimeter, and this has been validated in our previous study (Tokuyama et al. 2009). Energy expenditure was calculated from O_2_ consumption, CO_2_ production, and 24‐h urinary nitrogen excretion (Ferrannini 1988; Sato et al. 2011; Kobayashi et al. 2014).

#### TEF calculation

To compare the TEF estimates with those of Tataranni's method as the gold standard, morning TEF (4 h from 0800 h) was calculated using four methods: (A) Tataranni, (B) Schutz, (C) modified Schutz, and (D) our original method. To compare the TEFs among these different methods, the TEF during the entire waking period (15 h from 0800 h) was also calculated by methods B, C, and D, and the TEF is expressed as the relative value (i.e., % of caloric intake).

##### Tataranni's method

Tataranni's method (Tataranni et al. 1995) calculates the TEF as the difference in energy expenditure (ΔEE) between the fed and fasting conditions. In the present study, the ΔEE due to breakfast was calculated during the morning hours from 0800 h to 1200 h.

##### Schutz's original method

Schutz's original method (Schutz et al. 1984) calculates the TEF as an increase in the EE_0_ above the RMR. The EE_0_ was calculated from the relationship between energy expenditure and physical activity at that moment. The RMR was defined as the average energy expenditure before breakfast (0715–0745 h, while the subject was seated). The EE_0_ above the RMR was calculated for 4 h in the morning and during the 15 h of waking.

##### Modified Schutz's method

The modified method of Schutz subtracts the SMR instead of the RMR from the EE_0_ to calculate the TEF (Westerterp et al. 1999). The SMR was defined as the average energy expenditure during the sleep period (2300–0700 h), because the SMR changes after the onset of sleep (Katayose et al. 2009). The EE_0_ above the SMR was calculated for 4 h in the morning and during the 15 h of waking.

##### Our original method

The postprandial increase in the energy expenditure free from NEAT was derived from its linear regression on integrated physical activity (Weinert and Waterhouse 1998). First, the physical activity measured at 1‐min intervals was integrated over a variable moving window (0 to ±30 min) with a time lag (−20 to 20 min). The combination of the integration time and time lag that provided the highest correlation (*r*) between the energy expenditure and integrated physical activity was determined for each subject and time period (i.e., 30 min prior to breakfast [0715–0745 h], 4 h in the morning, and 15 h of waking period). Second, NEAT was estimated from its linear dependency of energy expenditure on integrated physical activity and increase in integrated physical activity above its lowest value. Third, NEAT was subtracted from total energy expenditure and a time course of energy expenditure free from NEAT was constructed. Finally, TEF was estimated as postprandial increase in energy expenditure free from NEAT. The preprandial metabolic rate was defined as the average energy expenditure free from NEAT before breakfast (0715–0745 h).

### Statistical analysis

Data are presented as the mean ± SD. The Pearson product–moment correlation coefficients between energy expenditure and (integrated) physical activity were calculated, and the differences in correlation coefficients between our original method (between energy expenditure and integrated physical activity) and Schutz's methods (between energy expenditure and physical activity) were evaluated using Student's *t* test. The breakfast TEF values were compared by one‐way analysis of variance (ANOVA) followed by a post hoc Bonferroni correlation calculation with a repeat number of three. The individual variability of breakfast TEF between the reference method and the other approaches was examined using the Bland–Altman procedure (Bland and Altman 1986). Differences in the magnitude of the TEF after each meal were evaluated by one‐way ANOVA followed by a post hoc Bonferroni test and Student's *t* test for the three‐ and two‐meal conditions, respectively. Differences in the energy metabolism and physical activity between the two dietary conditions were evaluated using Student's *t* test. All statistical analyses were performed using SPSS version 19.0 (SPSS Japan, Tokyo, Japan). The level of significance was set at *P *<* *0.05.

## Results

### Energy metabolism and physical activity

**Figure 1:**
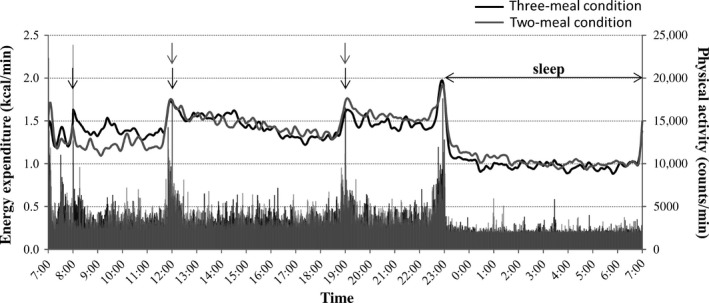
Mean diurnal changes in energy expenditure and physical activity in the three‐ and two‐meal conditions. Energy expenditure and physical activity are presented as line and bar graphs, respectively. Arrows indicate meal times. The TEF estimated by Tataranni's method, that is, ΔEE due to breakfast, is shown as the difference in energy expenditure between the two dietary conditions in the morning.

Between the three‐ and two‐meal conditions, there were no significant differences in the total energy expenditure (1865 ± 202 and 1876 ± 179 kcal/day, respectively) or average physical activity (3455 ± 487 and 3521 ± 469 counts/min, respectively) during 24 h. Diurnal changes in energy expenditure and physical activity are presented as the mean value for each dietary condition (Fig. 1). Mean energy expenditure and physical activity levels were higher while the subjects were awake compared to when they were asleep, and episodically increased when the subjects consumed meals, prepared to sleep, and got out of bed. Compared with the two‐meal condition, the energy expenditure in the three‐meal condition was significantly higher after breakfast (1.409 ± 0.19 and 1.244 ± 0.14 kcal/min for the three‐ and two‐meal conditions, respectively, 0800–1200 h, *P *<* *0.01), but significantly lower after dinner (1.508 ± 0.19 and 1.587 ± 0.17 kcal/min, 1900–2300 h, *P *<* *0.05) and during sleep (1.000 ± 0.09 and 1.049 ± 0.09 kcal/min, 2300–0700 h, *P *<* *0.05). Despite the significant difference in energy expenditure during sleep between the two dietary conditions, there were no unexpected increases in energy expenditure (see Fig. 1) or physical activity (2240 ± 119 and 2265 ± 82 counts/min) such as using the toilet and moving around the room.

### TEF estimated by Tataranni's method

The difference in morning energy expenditure between the two dietary conditions (i.e., ΔEE) from 0800 h to 1200 h was 5.4 ± 3.5% (Table 1).

**Table 1:** Estimated TEF according to four calculation methods

Condition	Three‐meal	Two‐meal
EE_0_ (kcal/min)^a^		
Morning	1.300 ± 0.206	
Entire waking period	1.366 ± 0.155	1.361 ± 0.213
Baseline (kcal/min)		
RMR	1.306 ± 0.300	1.247 ± 0.128
SMR	1.011 ± 0.089	1.038 ± 0.092
Preprandial energy expenditure	1.160 ± 0.191	1.092 ± 0.143
Breakfast TEF (% of breakfast energy content)		
(1) ΔEE	5.4 ± 3.5 [1.6–12.7]	
(2) EE_0_ above RMR^b^	−0.6 ± 6.8 [−13.1–5.9]	
(3) EE_0_ above SMR^b^	9.8 ± 5.7 [0.3–19.1]	
(4) Energy expenditure free from NEAT above preprandial value	4.1 ± 2.5 [0.3–7.4]	
TEF during waking^d^ (% of daily energy intake)		
(2) EE_0_ above RMR^c^	2.6 ± 8.7 [−16.0–9.0]	3.0 ± 4.8^d^ [−7.2–7.4]
(3) EE_0_ above SMR^c^	14.5 ± 2.5 [12.4–19.2]	9.5 ± 3.3 [3.0–13.2]
(4) Energy expenditure free from NEAT above preprandial value	6.8 ± 4.0^d^ [0.6–11.9]	7.7 ± 2.6 [3.4–11.5]

Data are mean ± SD [range]. The TEF is expressed as the % of energy intake during measurement. EE_0_, energy expenditure at 0 activity; ΔEE, the difference in energy expenditure between the fed and fasting states; RMR, the average energy expenditure before breakfast (0715–0745 h); NEAT, nonexercise activity thermogenesis; SMR, average energy expenditure during sleep (2300–0700 h); preprandial value, average energy expenditure free from NEAT before breakfast (0715–0745 h); TEF, thermic effect of food.

a The EE_0_ and the TEF during waking in the two‐meal condition were assessed over 11 h (1200–2300 h).

b TEF (%) = (EE_0_ in the morning − RMR or SMR)/energy intake × 240 min × 100.

c TEF (%) = (EE_0_ during the full waking period − RMR or SMR)/energy intake × 900 or 660 min × 100.

d Mean values significantly different from those of EE_0_ above SMR (*P *<* *0.05) determined by one‐way ANOVA followed by a Bonferroni post hoc test.

### TEF estimated by Schutz's methods

**Figure 2:**
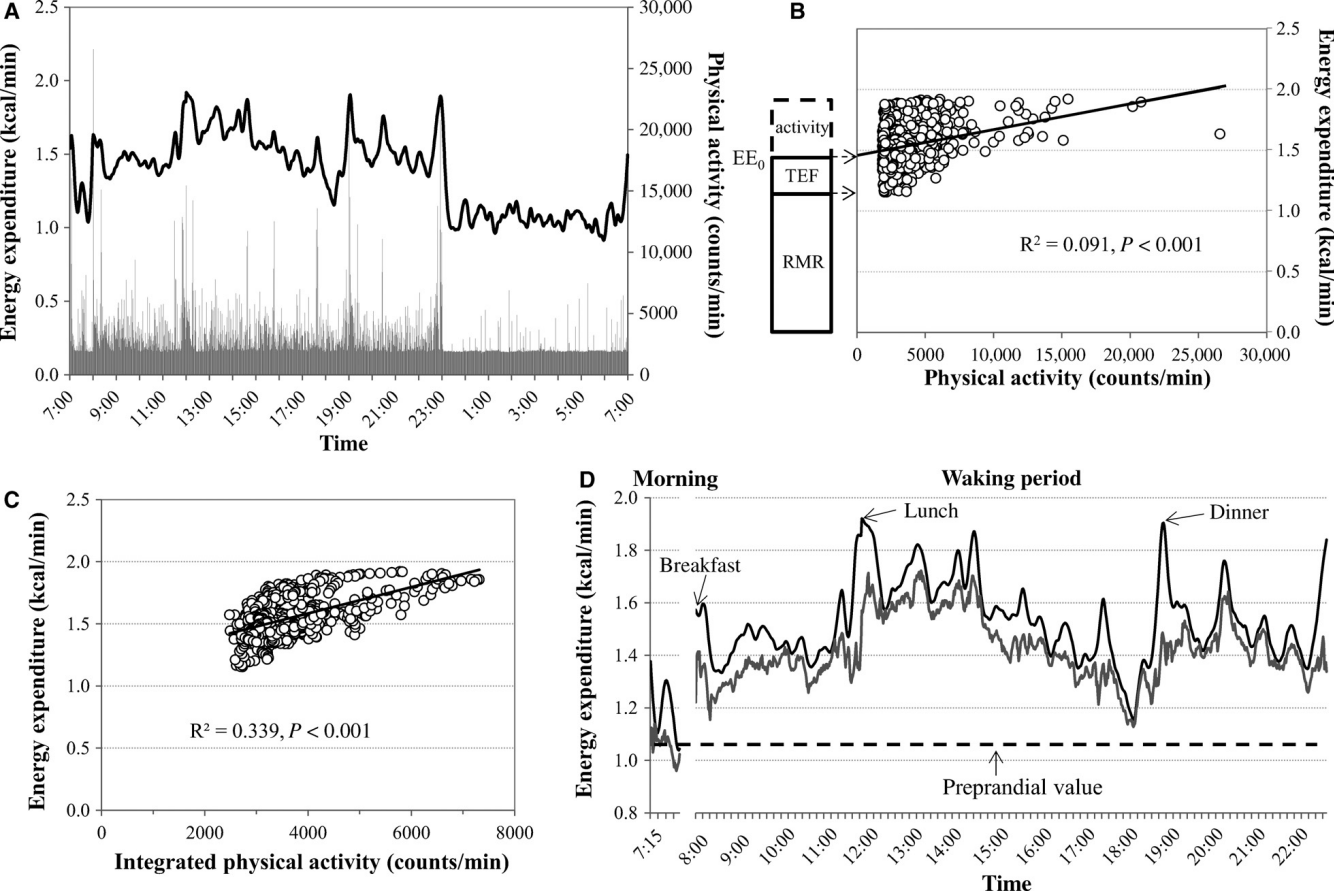
Simplified methods for TEF estimation. (A) Typical diurnal changes in energy expenditure (line graph) and physical activity (bar graph) in the three‐meal condition. (B) Relationship between energy expenditure and physical activity during the waking period after breakfast (0800–2300 h). In this case, the *y*‐intercept for the regression line, that is, energy expenditure at 0 activity (EE0), was 1.461 kcal/min. The RMR, calculated as the average energy expenditure before breakfast (0715–0745 h), was 1.195 kcal/min. In Schutz's original method, the TEF, calculated as the difference between EE0 and the RMR, was 240.1 kcal (9.0% of daily energy intake). (C) Correlation between energy expenditure and “integrated” physical activity during the waking period (0800–2300 h). In this case, the optimal integrated time ranges were ±11 min in the morning (0800–1200 h) and ±7 min during 15 h of waking (0800–2300 h). (D) Energy expenditure (black line) and its component free from NEAT (gray line). The correlations between energy expenditure and integrated physical activity were calculated for two separate periods and consequently there is a gap in energy expenditure before breakfast. Our original method for calculating the TEF, that is, energy expenditure free from NEAT above the preprandial value, was calculated as the difference between preprandial energy expenditure (1.061 kcal/min) and the time course of energy expenditure free from NEAT. In this example, the TEF accumulated over 15 h was 11.9% of caloric intake.

Typical diurnal changes in the energy expenditure and physical activity in a subject in the three‐meal condition are shown in Figure 2A. From the linear relationship between energy expenditure and physical activity during the waking period after breakfast (0800–2300 h), EE_0_ was obtained as the *y*‐intercept, and the TEF was calculated as the difference between the EE_0_ and the RMR or SMR (Fig. 2B). In this example, the EE_0_, RMR, and SMR were 1.461, 1.195, and 1.078 kcal/min, respectively. Hence, the 15‐h TEF, defined as the EE_0_ above the RMR, was 240.1 kcal (9.0%), and the TEF, defined as the EE_0_ above the SMR, was 344.9 kcal (12.9%).

The average EE_0_ values of the seven subjects in the three‐ and two‐meal conditions were 1.366 ± 0.155 and 1.361 ± 0.213 kcal/min, respectively. The estimated TEF, defined as EE_0_ above the RMR, during the 15 h waking period was 2.6 ± 8.7% and 3.0 ± 4.8% for the three‐ and two‐meal conditions, respectively; similarly, the TEF, defined as EE_0_ above SMR, during the 15 h waking period, was 14.5 ± 2.5% and 9.5 ± 3.3%, respectively (Table 1).

To compare the TEF estimates with those calculated from Tataranni's method, which was only applicable during the morning hours in the present study, the EE_0_ in the three‐meal condition was evaluated using data collected in the morning (0800–12:00 h). The average EE_0_ in the three‐meal condition was 1.300 ± 0.206 kcal/min, from which the RMR (1.306 ± 0.300 kcal/min) or SMR (1.011 ± 0.089 kcal/min) was subtracted to estimate the TEF. Hence, the estimated TEF after breakfast defined as the EE_0_ above the RMR and SMR was −0.6 ± 6.8% and 9.8 ± 5.7%, respectively (Table 1).

### TEF estimated by our original method

An example of the relationship between energy expenditure and integrated physical activity is shown in Figure 2C, from which the energy expenditure free from NEAT was calculated (Fig. 2D). The correlation between energy expenditure and the optimal integrated time ranges of physical activity (±7 min, *R* ^2^ = 0.339, Fig. 2C) was higher than that between energy expenditure and physical activity using Schutz's method (*R* ^2^ = 0.091, Fig. 2B). In this example, the accumulated TEF over 4 h in the morning and the whole 15 h waking period was 7.4% and 11.9%, respectively.

**Figure 3:**
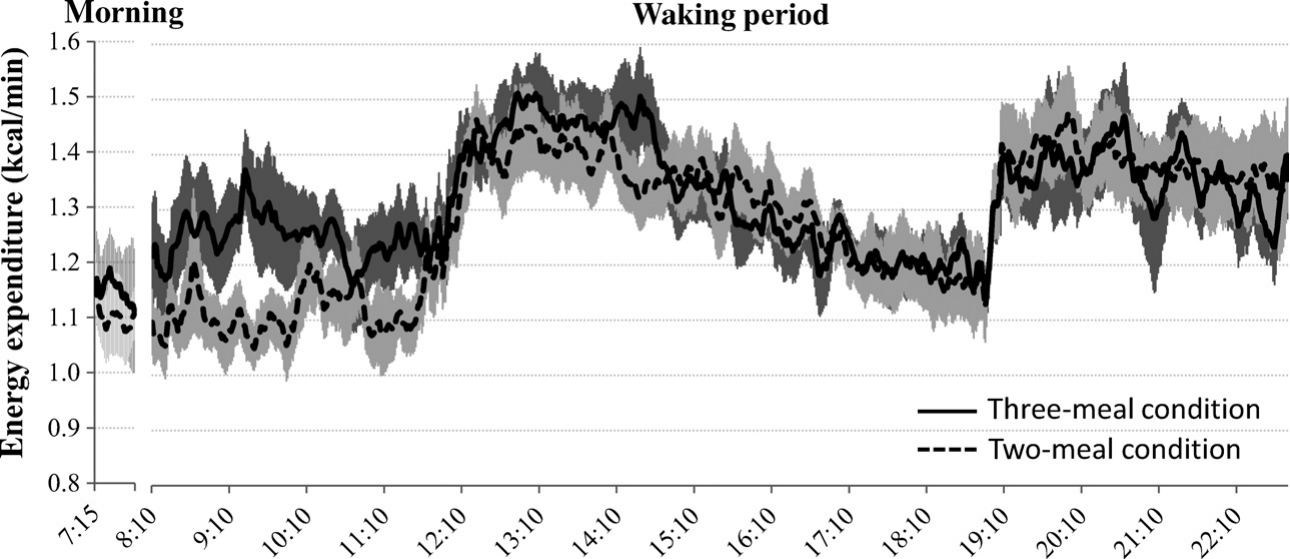
Mean diurnal changes in energy expenditure free from NEAT in the three‐ and two‐meal conditions. Each standard error of the mean is represented by shading. The correlations between energy expenditure and integrated physical activity were calculated for two separate periods and consequently there is a gap in energy expenditure free from NEAT before breakfast.

The correlation between energy expenditure and integrated physical activity during 15 h of waking (*R* ^2^ = 0.445 ± 0.038) in the seven subjects was significantly higher than that between energy expenditure and physical activity using Schutz's method (*R* ^2^ = 0.165 ± 0.025, *P *=* *0.001). The optimal integration time ranged from 3 to 11 min for the seven subjects and was unaffected by the number of meals. The mean lag time that produced the highest correlation between energy expenditure and integrated physical activity was 0.0 ± 1.5 min. NEAT during the 16 h of waking in the three‐ and two‐meal conditions was estimated to be 9.0 ± 0.9% and 10.6 ± 1.3% of total energy expenditure, respectively (*P *=* *0.12). For each dietary condition, the time courses of the energy expenditure free from NEAT are presented as mean ± standard error of the mean in Figure 3.

The morning TEF calculated by our method was 4.1 ± 2.5%. During the 15 h of waking, the TEF in the three‐ and two‐meal conditions was 6.8 ± 4.0% and 7.7 ± 2.6%, respectively (Table 1).

The accumulated TEF over 4 h after each meal was comparable in the three‐meal condition (4.1 ± 2.5%, 5.7 ± 3.6%, and 6.0 ± 3.7%, after breakfast, lunch, and dinner, respectively), whereas the TEF after dinner (6.6 ± 2.0%) was significantly higher than that after lunch (5.1 ± 1.8%) in the two‐meal condition (*P *<* *0.01) (Table 2).

**Table 2:** TEF during the 4 h after each meal estimated using method D: energy expenditure free from NEAT above the preprandial value

Condition	Three‐meal	Two‐meal
Breakfast TEF (% of breakfast energy content)	4.1 ± 2.5 [0.3–7.4]	
Lunch TEF (% of lunch energy content)	5.7 ± 3.6 [0.6–10.8]	5.1 ± 1.8^a^ [2.0–7.7]
Dinner TEF (% of dinner energy content)	6.0 ± 3.7 [0.4–10.3]	6.6 ± 2.0 [3.2–9.5]

NEAT, nonexercise activity thermogenesis; TEF, thermic effect of food.

Data are mean ± SD [range]. The TEF is expressed as % of energy intake.

a Significant difference between the TEF after lunch and after dinner (*P *<* *0.05) determined by Student's *t* test.

### Comparison of TEF estimated by different methods

**Figure 4:**
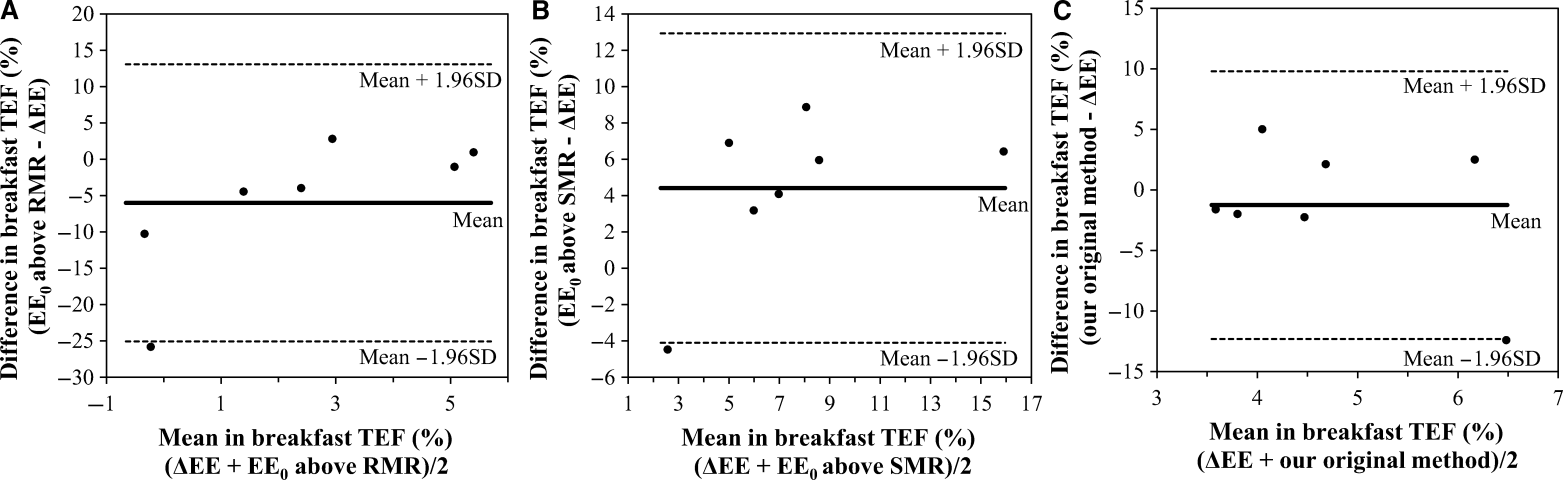
Bland–Altman plot of the individual variability of breakfast TEF between the reference method and the other approaches. The mean values (A, between ΔEE and EE0 above RMR; B, between ΔEE and EE0 above SMR; and C, between ΔEE and our original method, i.e., energy expenditure free from NEAT above the preprandial value) are plotted against the difference of the same two values. The solid line represents the mean difference and the dashed lines are the upper and lower limits of agreement (1.96 SD).

Regarding the estimated TEF for breakfast over 4 h in the morning, ΔEE, EE_0_ above the SMR, and accumulated energy expenditure free from NEAT above the preprandial value were positive in all cases. However, in the estimates based on Schutz's original calculation, the EE_0_ above the RMR returned negative values in three cases. The EE_0_ above the SMR was higher than the ΔEE, although this difference was not significant (*P *=* *0.11). The Bland–Altman plot of the individual variability of breakfast TEF between the ΔEE and the other approaches are shown in Figure 4. The mean difference between ΔEE and EE_0_ above RMR was −5.989% (Fig. 4A), between ΔEE and EE_0_ above SMR was 4.414% (Fig. 4B), and ΔEE and our original method was −1.257% (Fig. 4C).

In a similar comparison among the three simplified methods, the average estimates of the TEF during 15 h of waking in the three‐meal condition and 11 h in the two‐meal condition in ascending order were EE_0_ above the RMR, our original method, and EE_0_ above the SMR. Furthermore, EE_0_ above the SMR was significantly higher than in our original method in the three‐meal condition and EE_0_ above the RMR was significantly lower than in EE_0_ above the SMR in the two‐meal condition (Table 1).

## Discussion

Various simplified methods for TEF estimation that do not require two separate measurements in fed and 24‐h fasted states have been proposed during the past three decades (Capani et al. 1984; Schutz et al. 1984; Segal et al. 1985; Ravussin et al. 1986; Weststrate et al. 1989; Romon et al. 1993; Tataranni et al. 1995; de Jonge and Bray 1997; Westerterp et al. 1999). However, such methods often produce negative values as TEF estimates (Ravussin et al. 1986; Westerterp et al. 1999). Therefore, we propose a novel method for the calculation of the TEF that involves integrated physical activity over a specific time interval. The present validation study compared three simplified TEF estimation methods against the method of Tataranni et al. (1995) as the gold standard. In particular, the present study aimed to answer two methodological questions: (1) Are all estimates of the TEF positive values? (2) Which simplified method returns a value similar to that of Tataranni et al.?

### Rationale for our TEF estimation method

The optimal integration time, that is, integrated physical activity, ranged from 3 to 11 min in the present study in which exercise was not performed during the calorimetry. The optimal integration time might be bigger when the subjects performed moderate‐ and/or higher intensity exercise. Time lag due to methodology using whole‐body indirect calorimetry was estimated as 2 min from intermittent gas infusion test (Tokuyama et al. 2009). In addition, it was plausible to assume a physiological time lag between changes in physical activity and energy expenditure. However, the mean physiological time lag was 0 min. Our system of indirect calorimetry seemed to be not sensitive enough to detect it. NEAT is the energy expended as a result of everything humans do that is not sleeping, eating, or performing sports‐like activities. Although subjects can be asked not to perform exercise, it is impossible to entirely suppress NEAT during the calorimetry. Herein, we propose a novel method to estimate the TEF by removing NEAT from the time course of energy expenditure. Physical activity is not immediately reflected as changes in energy expenditure because of the oxygen deficit at the onset of exercise and excess postexercise oxygen consumption (LaForgia et al. 2006). Similarly, the relationship between physical activity and body temperature is not straightforward, and changes in body temperature reflect the integrated activity, that is, the accumulated activity over a given time interval (Weinert and Waterhouse 1998). It is therefore reasonable to expect that the time course of energy expenditure is more closely related to integrated physical activity than physical activity. In the present study, we proposed isolating NEAT, which is derived from the linear dependence of energy expenditure on integrated physical activity, and thus defined the TEF as the postprandial increase in energy expenditure free from NEAT. We measured physical activity using a triaxial accelerometer, and the average activity level (~3500 counts/min) roughly corresponded to the levels associated with normal activities (i.e., 8.4 mG = 3500 counts/min × 2.4 *μ*G/count) such as resting in a sitting position and working on a personal computer (Ohkawara et al. 2011). The total NEAT during the 16 h of waking was approximately 10% of the total energy expenditure for the two dietary conditions, corresponding to the subject's sedentary state.

### Comparison of TEF estimates from different methods

The method of Tataranni et al. (1995) for comparing energy expenditure between fed and fasted states was only applicable during the morning hours (0800–1200 h), and the ΔEE during this period was 5.4 ± 3.5% of the breakfast energy content. All estimates were positive, but the average value was slightly less than previously reported (Westerterp 2004; Levine 2005), probably because of the short time period for assessing the TEF after breakfast. Although the mean EE_0_ above the RMR was not significantly different from ΔEE (*P *=* *0.47), the estimates were negative in three out of seven cases. Therefore, the SDs of the TEF estimation was higher than for the other calculation methods. In the present study, the RMR was defined as the energy expenditure before breakfast while the subject was sitting, which is higher than the metabolic rate measured while the subject is lying in a supine position, that is, the BMR. We considered this to be an appropriate method for calculating the TEF, because the TEF should reflect the increase in metabolism resulting from the ingested meal rather than a change of posture. The estimated EE_0_ above the SMR was slightly higher than the ΔEE (*P *=* *0.11) and was positive in all cases. If the SMR is defined using the same criteria that were described in previous studies, that is, the average minimum energy expenditure during three consecutive hours of sleep (Schrauwen et al. 1997; Westerterp et al. 1999; Ganpule et al. 2007; Smeets and Westerterp‐Plantenga 2008) and/or the lowest energy expenditure during three consecutive hours with minimal movement (Westerterp‐Plantenga et al. 2002), the EE_0_ above the SMR might be significantly higher than the ΔEE. Our original method returned values close to ΔEE (*P *=* *0.50), which were positive in all cases. Although the present study had a small sample size, we performed a power analysis with *α* = 0.0167, and the actual power was more than 80% for each comparison. When the individual variability of breakfast TEF between the ΔEE and the other approaches were examined, the Bland–Altman plot only showed that the mean of the difference in ΔEE and our original method, that is, energy expenditure free from NEAT above the preprandial value, fell close to the zero line. These results indicated that there was no bias and therefore in general the two methods were producing the same results.

When the TEF during 15 h of waking was estimated, the EE_0_ above the RMR was negative in three out of 14 cases, whereas all estimates using the EE_0_ above the SMR and using our original method were positive. The average estimates of the TEF from the three methods in both dietary conditions in ascending order were as follows: EE_0_ above the RMR, our original method, and EE_0_ above the SMR. Compared with each SD in the same dietary and measurement condition, the SD of our original method was generally lower among the calculation methods, and we therefore conclude that our original calculation provides a robust and highly reproducible TEF estimation.

### Time course of TEF by our original calculation

It is noteworthy that the method proposed herein enables the evaluation of the TEF time course, which is a potential advantage owing to the multicomponent nature of TEF, whereas Schutz's methods provide an average value during the experiment (Schutz et al. 1984; Westerterp et al. 1999). Visual inspection of the energy expenditure free from NEAT shows that it did not return to the preprandial value before lunch (4 h after breakfast) or bedtime (4 h after dinner). Therefore, the TEF during 4 h in the morning and evening might underestimate the total TEF for breakfast and dinner; this is consistent with previous studies on the time course of energy expenditure intermittently measured after a meal (Belko et al. 1986; D'Alessio et al. 1988; Kinabo and Durnin 1990; Reed and Hill 1996). Despite the possible underestimation of the TEF after breakfast and dinner, accumulated TEF was highest during the 4‐h after dinner in the three‐meal condition, followed by lunch and breakfast. Similarly, in the two‐meal condition, the TEF after dinner was significantly higher than that after lunch. Thus, within 1 day, the TEF after dinner was higher than that after other meals. It is also notable that the preprandial interval differed among meals: 13, 4, and 7 h before breakfast, lunch, and dinner, respectively, in the three‐meal condition. Therefore, further studies are required to clarify the factors determining the magnitude of the TEF.

### Limitations

This study has a number of limitations. First, our original method for TEF estimation could not be applied during the sleeping period, because the energy cost of arousal was not taken into account. However, our observations suggest the TEF persists during sleep. Second, the RMR was measured in a sitting position instead of a supine one, and its relatively high value compared to the BMR might have contributed to the negative values obtained using Schutz's method. Third, the subjects in the present study were normal weight. However, TEF of obese subjects could be estimated as the same calculation method, since the positive correlation between physical activity and energy expenditure is individually estimated. Finally, physical activity in the chamber was restricted. After entering the chamber, the subjects remained sedentary, except for example when they took and returned meal plates, changed clothes before and after sleep, and used the toilet. As the magnitude of excess postexercise oxygen consumption increases exponentially with increasing exercise intensity (Børsheim and Bahr 2003), the simple linear regression between energy expenditure and integrated physical activity used in the present study might have to be modified, that is, the multiple equation models, if higher intensity exercise is included in the experiment.

## Conclusions

### Are all estimates of the TEF positive?

Whether all estimates of the TEF are positive is of prime importance because negative values of the TEF are inconsistent with its definition. The ΔEE, EE_0_ above the SMR, and our original method satisfy this requirement, but negative estimates were observed in some cases for the EE_0_ above the RMR.

### Which simplified method is ideal for TEF estimation?

Our original method, in which NEAT is removed from the time course of energy expenditure, is the best among the three alternative methods according to two criteria: returning positive estimates of the TEF in all measurements and returning estimates close to the ΔEE.

## Conflicts of Interest

None declared.
